# Establishing a clinically applicable frailty phenotype screening tool for aging dogs

**DOI:** 10.3389/fvets.2024.1335463

**Published:** 2024-09-25

**Authors:** Katharine J. Russell, Alejandra Mondino, Gilad Fefer, Emily Griffith, Korinn Saker, Margaret E. Gruen, Natasha J. Olby

**Affiliations:** ^1^Department of Clinical Sciences, College of Veterinary Medicine, North Carolina State University, Raleigh, NC, United States; ^2^Department of Statistics, College of Sciences, North Carolina State University, Raleigh, NC, United States

**Keywords:** frailty, canine frailty, frailty phenotype, geriatric, aging dogs

## Abstract

**Introduction:**

Frailty is a well-defined clinical syndrome in humans caused by accumulation of impairments which result in loss of reserve capacity and increased vulnerability to disability, dependence, and death. Dogs are of particular interest in studies of frailty due to the similarities they share with people in their environment, lifestyles, and age-related diseases.

**Materials and methods:**

The aim of this study was to develop a frailty phenotype screening tool, based on previously validated measures in dogs, which could be easily applied in the clinical setting, and which was predictive of all-cause, short term (6-month) mortality. The study was conducted in two phases. In phase 1, a retrospective cohort of 51 dogs was used to identify and evaluate potential measures for the five domains of frailty. This information was then used to develop a simple frailty phenotype based on examination findings and owner directed questions. In phase 2 of the study, this phenotype was evaluated in a prospective cohort of 198 dogs aged 9 years or older from multiple different specialty and primary care services to determine how the phenotype performed across a diverse canine population.

**Results:**

The developed frailty phenotype was predictive of all-cause, short-term mortality independent of age, sex, or weight (hazard ratio = 4.71; 95% CI, 2.66–8.8). Of the covariates evaluated only breed was significant, with purebred dogs having 1.85 times higher mortality than mixed breed dogs (95% CI, 1.04–3.31). The frailty phenotype performed similarly across all hospital services from which patients were enrolled.

**Conclusion:**

Based on these findings, the defined frailty phenotype represents a valuable screening tool for early risk identification and intervention, and can aid in clinical decision making for owners and veterinarians. Additionally, it will promote further research into the understanding and treatment of frailty in dogs.

## 1 Introduction

Frailty is defined as an accumulation of impairments which result in loss of reserve capacity and consequently an increased vulnerability to disability, dependence, and death (1). The concept of frailty as a defined clinical syndrome affecting elderly people was first proposed in a pivotal study by Fried et al. (2). They identified a frailty phenotype in people that captures the complex interplay of factors which result in erosion of physiological reserve. It predicts mortality and allows identification of affected domains and areas of focus for targeted therapies to slow development or progression of frailty and reduce the risk of disability, hospitalizations, and death in at-risk populations. The pathophysiology of frailty is made up of a complex interplay of biological, systemic, and functional impairments and imbalances leading to the vicious cycle of frailty. Among these mechanisms include factors such as increasing oxidative stress, chronic inflammation, mitochondrial dysfunction, decreased blood perfusion, loss of regenerative potential of stem cells, hormonal imbalances, influences of chronic disease states, and many other factors that change the homeostatic balance within the body. This interplay of impairments leads to the compromised response to stressors and vulnerability that we associate with frailty (3).

As the clinical interest in frailty has grown, different methods of identifying and quantifying frailty have been proposed and validated in people. The two most commonly referenced models for frailty include a model of “phenotypic frailty” and a “deficit accumulation model” (4, 5). The phenotypic model of frailty identifies frailty as a unique clinical syndrome which occurs independent of other age associated morbidities (4). Frailty phenotypes require patients to self-report symptoms and involve simple, readily deployable tests of strength and walking speed to capture the clinical consequences of this age-associated syndrome. In contrast, the deficit accumulation model seeks to generate a granular scale by summing multi-dimensional measures of patient health. They use measures such as number of comorbidities and medications as well as changes in blood work, other biomarkers, and social and physical impairments to form a composite index for prediction of risk. As such, these two approaches have rather different goals but are complementary.

Studies comparing frailty phenotypes and indices have found that both predict disability, morbidity, and both short-and long-term mortality but that each measure has its own strengths and weaknesses. The frailty phenotype is composed of a few simple measures which are based largely on clinical signs, many of which can be identified before a clinical assessment has even occurred. These clinical signs may be associated with, or could occur independent of, other age associated comorbidities and the measure does not directly factor in specific diseases. The broad measures of a phenotype allow it to be used as a point-of-care test for at-risk individuals, and to identify impairments which may be therapeutically targeted. However, being limited to a few broad measures means the frailty phenotype lacks some of the granularity of the index (6). The frailty index, in contrast, is based on the presence or absence of specific disease states, laboratory abnormalities, physical and social impairments, and other similar measures. These measures mean that it requires a comprehensive clinical assessment. With its numerous measures, it is superior at detecting smaller fluctuations in frailty severity but generally requires a more extensive history and diagnostic approach to administer (6). The frailty measure that a researcher or clinician employs is often determined by personal preference or based on which measure can best address available data and in many instances frailty phenotypes and indexes are complementary, allowing each to balance the other’s weaknesses. Longitudinal studies of frailty have also found that the components of frailty are dynamic, and their states can change over time and with intervention (4, 7, 8). This makes targeting and intentionally reversing frailty feasible.

As the interest in measuring frailty in people has grown, so too has the interest in measures of frailty in animals. Frailty measures have been described in multiple non-human species including mice, rats, nematodes, and dogs (9–12). Companion dogs are of particular interest in aging research due to the similarities with people in shared environments and lifestyles. They also share many of the same age-related diseases and age-associated cognitive impairments as humans (13). At the time of writing, two frailty phenotypes and an index have been described for companion dogs (10, 12, 14). These measures of frailty established the presence of a frailty syndrome in dogs analogous to human frailty. The canine frailty phenotypes defined criteria for the five domains described in people and the resulting phenotype was associated with mortality but included criteria that we found challenging to apply consistently such as hair coat quality (10). The published canine index, in contrast, consisted of 33 different components and predicted short term mortality with moderate accuracy but required considerable medical testing that was not readily available for our population of dogs (12). We wanted to develop a tool which could be deployed across all types of veterinary clinics and all dog breeds, acting as a rapid screening tool for frailty without requiring an extensive workup. The objective of this study was to develop and evaluate a measure of frailty for companion dogs of all breeds that could be easily applied without extensive medical testing, and that was highly predictive of short-term (6 month) mortality in a wide range of different clinical settings.

## 2 Materials and methods

All study protocols were conducted with the approval of the North Carolina State University (NCSU) Institutional Animal Care and Use Committee (protocol numbers 21-303 and 21-381). All procedures were performed in accordance with these approved protocols and institutional guidelines. Owners of the dogs who participated in these studies reviewed and signed an informed consent form. Methods were carried out in accordance with relevant guidelines and regulations and reported in accordance with ARRIVE guidelines.^1^ IRB approval was not sought because all collected data pertained to dogs and as such the work was categorized as “Non-Human Subject Research.” All decisions regarding euthanasia were made by the owners and were independent of this study or its findings. The work was carried out in 2 phases.

### 2.1 Phase 1: Frailty phenotype development

#### 2.1.1 Animals

Population 1 of the study included 51 dogs enrolled in a longitudinal study of neuro-aging at the NCSU College of Veterinary Medicine as detailed in Fefer et al. (15). All dogs in the study were in the last 25% or beyond their expected lifespan per AKC breed standards. Dogs were excluded from the study if they had any comorbidities that would preclude cognitive testing such as blindness or an inability to walk independently. Dogs with comorbidities commonly seen in advanced age that did not interfere with cognitive testing, such as osteoarthritis or stable chronic kidney disease, were not excluded. All dogs in the study underwent regular evaluations spaced 3–6 months apart and were followed from the time of enrollment until they died or were lost to follow-up. Dogs were selected for inclusion in the frailty study if their existing data were sufficient to assign a score for individual components of frailty, as defined in the following sections, and had either died or were at least 6 months out from the time of enrollment.

#### 2.1.2 Data collection and defining the frailty phenotype

Demographic data were collected for each dog and the owners were asked to complete a series of questionnaires for each visit that captured owner assessments of appetite, mobility, pain, cognition and continence. The dogs were evaluated physically, orthopedically, and neurologically and were assigned muscle condition scores (MCS) and body condition scores (BCS) as part of this assessment. The MCS and BCS were based on a 3-point and 9-point scale, respectively (16) (Supplementary Data Sheet 1).

Five domains are recognized in phenotypic studies of frailty in humans including unintentional weight loss (loss of >10 pounds in 1 year), self-reported exhaustion, low physical activity (measured as kcal expended per week), weakness (measured by hand grip strength), and slow walking speed (4). These components have been used in a number of other human studies with minor adjustments. As not all of the above measurements of frailty can be feasibly evaluated in dogs, roughly equivalent measures were considered. Hua et al. (10) defined and validated a canine frailty phenotype approximately equivalent to that used in people but modified the domains slightly to include chronic undernutrition (based on body condition, changes in appetite, and hair coat quality), exhaustion (based on fatigability or marked breathlessness), weakness (based on muscle condition), low physical activity (based on the dog’s perceived activity level), and poor mobility (based on gait abnormalities, and the presence or absence of joint pain). These items were scored as present or absent by the veterinarian during their assessment of the patient.

We used the human and canine validated phenotypes as a guide to identify questionnaire and physical examination data from the longitudinal aging study which were roughly equivalent for each domain. Unlike the previous canine study, we drew heavily on owner evaluation of their dog’s activity, exhaustion, nutritional status and mobility, in recognition of the fact that companion dogs are often euthanized as their quality of life deteriorates, and owners make that decision. Each measure was evaluated in relation to 6-month mortality to determine its effectiveness as a measure of frailty. The five domains were identified as: nutritional status, exhaustion, muscle weakness (representing weakness), social activity level (representing activity level), and mobility (representing slow walking speed). Potential components of each domain of frailty were initially evaluated qualitatively. The data (questionnaire responses or physical examination findings) were assigned ordinal scores and graphed as scatter plots with dogs classified as either alive or dead within 6 months. Measures that appeared to discriminate between these two groups of dogs were kept for further analysis, while those that did not were discarded. Cutoffs were proposed for the measures that were retained which best visually discriminated between the two classifications. These thresholds were used to classify the dog as either impaired or not-impaired in each domain and advanced to further statistical analysis.

### 2.2 Statistical analysis

Statistical analyses were performed using RStudio (Build 492 RStudio, PBC, Boston, MA) and JMP Pro, (Version 15.2.0., SAS Institute Inc., Cary, NC).

For each retained measurement, the data were arranged in an ordinal fashion and the different cutoffs were evaluated visually using Kaplan–Meier survival curves allowing us to visually study how the measures behaved in relationship with mortality over the 6-month period. This evaluation helped to identify measures that did not perform as expected or changed in character over time and further narrowed the selection of viable measures. Receiver operating characteristic (ROC) curve analyses were then performed to evaluate the proposed cutoffs for these measures with an eye towards their clinical use. They helped to reaffirm the optimal cutoff for predicting 6-month mortality and evaluate the sensitivity, specificity, positive predictive value, negative predictive value, and accuracy of that cutoff. The measures with the best sensitivity and specificity were further evaluated using Cox proportional hazard analysis to examine each measure’s hazard level and whether each measure performed similarly to other measures. Finally, these measures were used to create an overall phase 1 frailty phenotype whose behavior was further evaluated in a similar fashion using Kaplan–Meier survival curves for visual analysis, ROC curve analysis to evaluate the optimal cutoff for overall frailty, and Cox proportional hazard analysis to evaluate risk associated with being frail. Some of the better performing measures that were excluded in this final step, were included in the questionnaire and data collection of Phase 2 in the event that any of the primary measures did not perform as expected.

Kaplan–Meier survival curves and univariate and multivariate Cox proportional hazard analyses were performed for each domain of the frailty phenotype, total impaired domains (the number of domains a dog was impaired in), and overall frailty (present or absent) to assess the significance of each in relation to 6-month mortality. Covariates were included to adjust for age only. This was elected in this predominantly exploratory phase of the study in order to avoid over interpretation of the measure which likely would be altered in phase 2 of the study. Time 0 was defined as the date at which the dogs underwent their evaluation for a particular visit. Time 1 was the number of days between Time 0 and death or 183 days whichever occurred sooner. The status of the dog (alive or deceased) was based on the status at 183 days following Time 0, and all dogs who were alive at 183 days from Time 0 were censored at that time. A unique ID was used to identify each dog, and was included as a random variable in the Cox proportional hazard analyses where more than one visit from a single dog were included. Statistical significance was defined as a *p* < 0.05.

### 2.3 Phase 2: Evaluation of the frailty phenotype

#### 2.3.1 Animals

Population 2 of the study included 198 client-owned dogs aged 10 years or older (or 9 years and older for giant breed dogs), who presented to the NCSU College of Veterinary Medicine Oncology, Internal Medicine, Cardiology, Neurology, Rehabilitation, or Small Animal Primary Care services. Dogs were also enrolled from a local general practice (Falls Village Veterinary Hospital). Dogs were excluded from the study if data for their signalment or any of the frailty domains at the time of enrollment were incomplete or if the dog’s status at 6 months could not be determined.

#### 2.3.2 Data collection

Demographic data were collected for each dog including breed, sex, date of birth, and whether age was known or estimated. Each component of the proposed frailty phenotype was incorporated into a short questionnaire and examination form. An additional question was included for each domain to allow an alternative to the original in the event any question performed poorly in population 2. The owners were asked to complete the questionnaire about their dogs’ appetite and diet, mobility, social activity level, the number of days per week they were anxious, and level of exhaustion. Weight, BCS, and MCS of the forelimbs, hindlimbs, and epaxial muscles were collected during the dogs’ routine physical examination. All owners were contacted 6 months following enrollment in the study to determine whether the dog was still alive, and if the dog had been hospitalized at any point since enrollment. For any owner who could not be contacted, records were reviewed to determine whether the dog was alive or deceased at 6 months. Of the 198 dogs in the study, 186 had completed follow-up surveys, the remaining 12 had their status determined through their medical records. If the dogs had died, their date and cause of death were also recorded (Supplementary material). See Supplementary material for the questionnaire and scoring guide used for each population.

### 2.4 Statistical analysis

All statistical analyses were performed using JMP Pro, Version 15.2.0. or Version 16.0.0. SAS Institute Inc., Cary, NC. ROC curve analyses using 5-fold cross validation were performed for each measure to determine if adjustments in the previously defined cutoffs remained the most appropriate and evaluate the sensitivity, specificity, positive predictive value, negative predictive value, and accuracy of the cutoffs in this population. Kaplan–Meier survival curves and univariate and multivariate Cox proportional hazard analyses were performed for each domain of the frailty phenotype, total impaired domains (the number of domains a dog was impaired in), and overall frailty (present or absent) to assess the significance of each in relation to 6-month mortality. Covariates were included to adjust for age, breed, sex, and weight. Intact or neutered status was not included in the analysis due to low numbers of intact dogs in the population. Time 0 was defined as the date at which the owners completed the enrollment questionnaire. Time 1 was the number of days between Time 0 and death or 183 days whichever occurred sooner. The status of the dog (alive or deceased) was based on the status at 183 days following Time 0, and all dogs who were alive at 183 days from Time 0 were censored at that time. No dogs from this population had repeat measurements. Statistical significance was defined as a *p* < 0.05.

## 3 Results

### 3.1 Phase 1: Frailty phenotype development

Of the 51 dogs in population 1, 39 had sufficient data to be included in the study. Of these dogs’ visits, 41% were spayed females, and 59% were neutered males. There were no intact dogs in this population. The median age at Time 0 for all visits was 12 years (range 9–17 years). Represented breeds included Labrador retriever (6), beagle (3), border collie (2), American Staffordshire terrier (2), (1), golden retriever (2), cairn terrier (2), Pembroke Welsh corgi (1), German shepherd (1), Jack Russell terrier (1), dachshund (1), Bernese mountain dog (1), Siberian husky (1), German shorthaired pointer (1), Brittany spaniel (1), great Dane (1), Irish setter (1), and the remainder were mixed breed dogs (12). Of the 39 dogs, 12 (30.8%) died over the evaluated period of time. Causes of death included euthanasia (10 dogs; 83.3%) due to neoplasia (3), unknown reasons (3), generally poor quality of life (2), chronic kidney disease (1), chronic respiratory disease (1); and natural causes (2 dogs; 16.7%) due to a foreign body airway obstruction (1), and snake envenomation (1) (Table 1).

**Table 1:** Association of demographic data and frailty.

		Population 1			Population 2		
		% of visits (*n* = 51)	Not frail (*n* = 28)	Frail (*n* = 23)	% of population (*n* = 198)	Not frail (*n* = 115)	Frail (*n* = 83)
Age	9 years	2%	1	0	0%	0	0
	10 years	15.7%	6	2	14.7%	22	7
	11 years	23.5%	11	1	26.2%	32	20
	12 years	13.7%	3	4	25.8%	31	20
	13 years	13.7%	3	4	11.6%	10	13
	14 years	13.7%	4	3	9.1%	10	8
	15 years	13.7%	0	7	8.6%	6	11
	16 years	2%	0	1	4%	4	4
	17 years	2%	0	1	0%	0	0
Breed	Purebred	76%	23	16	56%	62	49
	Mixed breed	24%	5	7	44%	53	34
Sex	Male	43%	16	6	50.5%	56	44
	Female	57%	12	17	49.5%	59	39
Small (<14.8 kg)		31%	7	9	48.5%	59	37
Med (14.9–24.9 kg)		28%	10	4	18.7%	24	13
Large (>24.9 kg)		41%	11	10	32.8%	32	33

A summary of demographic data for populations 1 and 2 with incidence of frailty listed for each population by measure. Note that dogs in population 1 had multiple visits and therefore numbers reflect the demographics for the visits. Percentages for population 1 are based on each visit, with some dogs being represented multiple times. Measures include age in years, purebred or mixed breed status (recorded for population 2 only), sex (includes but intact and neutered dogs under one heading), and weight by category (small, medium, or large).

The final measures identified for each domain of frailty for phase 1 are as follows: abnormal nutritional status was defined as a BCS below normal (<4 on a scale of 1–9) or a reduced appetite based on owner questionnaires. Exhaustion, social activity level, and mobility were also scored based on owner questionnaires. Exhaustion was based on a single question from the Liverpool Osteoarthritis in Dogs (LOAD) questionnaire, and asked the owners to score how frequently their dog stopped to rest during exercise [never, hardly ever, or occasionally (not impaired) vs. frequently or very frequently (impaired)]. Social activity level was based on a question from the longitudinal aging study’s enrollment survey which asks the owner to rank their dog’s engagement and activity around the house [plays and interacts normally (not impaired), vs. slight reduction in interaction, no longer getting up when the owner gets home, not wanting to play with their favorite toy, or showing no interest in stimuli (impaired)]. Mobility was based on the mobility section of the longitudinal aging study’s enrollment survey which asked the owner to note the frequency of weakness, lameness, stiffness, and mistakes made (scuffing, crossing over, skipping, etc.) while walking. Each mobility question was scored and the sum of the scores was used to determine the presence or absence of frailty in that domain with a minimum score of 0 (no abnormalities) and a maximum score of 7 (gait abnormalities noted >50% of the time) [sum of scores <5 (not impaired) vs. sum of scores ≥5 (impaired)]. Muscle condition was based on the MCS of the epaxial muscles [normal to moderate muscle atrophy (not impaired) vs. severe muscle atrophy (impaired)]. MCS of the hindlimbs was also evaluated and correlated well with aging and mortality but epaxial muscles are considered to be an earlier indicator of sarcopenia in dogs and so was considered preferential to the hind limbs or forelimbs (17, 18). The results of each component were reported as either 0 (not impaired) or 1 (impaired) and summed to determine their total impaired domains. Based on previous literature and the statistical analysis, a dog was considered frail if their total number of impaired domains was ≥2 and non-frail if <2.

The number of visits each dog had over the course of the evaluated time ranged from 1 to 5 with a total of 82 visits across all dogs. Completeness of information varied between visits due to questionnaire refinement and addition of new questionnaires in the longitudinal study over time. Dogs who were enrolled early in the study had fewer questionnaires they were expected to complete. Of the 82 visits, 82 (100%) had data for nutritional status, 76 (92.7%) for social activity, 43 (52.4%) for exhaustion, 70 (85.4%) for muscle condition, and 42 (51.2%) for mobility. Of the 82 visits, 7 (8.5%) had data for all five domains, 56 (68.2%) had data for four domains, 16 (19.5%) had data for three domains, and 4 (4.9%) had data for only two domains. Of the 82 visits across all dogs, 51 visits (62.2%) had sufficient information to definitively classify a dog as frail or non-frail for that visit based on an overall frail cutoff of two or more domains. Of those visits, 23 dogs were classified as frail (45%) and 28 were non-frail (54.9%). Of those frail dogs, 10 died within 6 months (43.5%); and of the non-frail dogs, one died within 6 months (3.6%).

**Figure 1:**
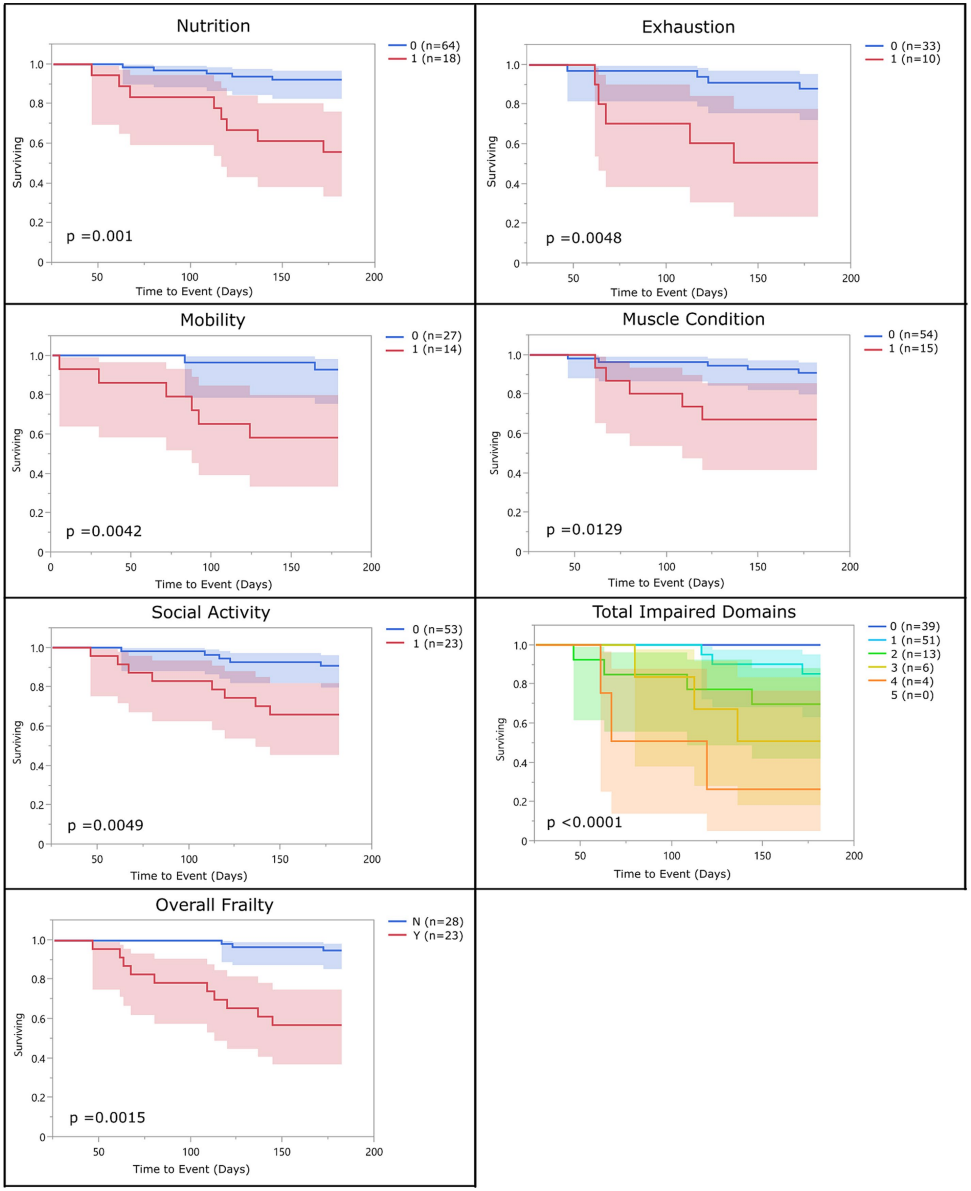
Survival curve by domain (population 1): population 1 survival curves representing the 6-month survival for each frailty domain, total impaired domains, and overall frailty. For each of the frailty domains (nutrition, exhaustion, mobility, muscle condition, and social activity), the blue line (0) represents the dogs who did not meet the criteria to be considered impaired within that domain and the red line (1) represents the dogs who did. Each dog was assigned a total impaired domains score based on the number of domains they met the criteria for (range 0–5). Dogs were classified as overall frail (Y) if they met the criteria for 2 or more domains, and non-frail (N) if they met the criteria for one or none of the domains.

For each domain, sensitivity ranged from 25 to 77% and specificity ranged from 76 to 97% (Table 2; Supplementary Data Sheet 2). All domains were significantly associated with 6-month mortality (*p* < 0.05), with hazard ratios ranging 4.25–7.38 (Figure 1; Table 3). Total impaired domains was significantly associated with 6-month mortality (*p* < 0.001) with a hazard ratio of 2.7 per unit (95% CI, 1.78–4.09) (Figure 1; Table 3). Overall frailty was also significantly associated with 6-month mortality (*p* = 0.001) with a hazard ratio of 8.014 (95% CI, 1.75–36.68) (Figure 1; Table 3) and predicted 6-month mortality with a sensitivity of 77% and specificity 81% (Table 2).

**Table 2:** Receiver operating characteristic curve analysis (Population 1).

Domain/measure	Optimal cutoff	AUC	Sensitivity	Specificity	PPV	NPV	Accuracy
Nutritional status (BCS)	3	0.59	25%	97%	0.6	0.88	0.86
Nutritional status (appetite)	3	0.72	62%	85%	0.47	0.91	0.81
Exhaustion	3	0.8	56%	85%	0.5	0.88	0.79
Mobility	5	0.79	75%	76%	0.43	0.93	0.76
Muscle condition	3	0.59	38%	86%	0.57	0.9	0.87
Social activity levels	2	0.71	62%	76%	0.35	0.91	0.74
Total impaired domains	2	0.88	77%	81%	0.44	0.95	0.8
Overall frailty	1	0.79	77%	81%	0.44	0.95	0.8

Results of the receiver operating characteristic (ROC) curve analyses for population 1. The ROC curve analyses were performed to reaffirm the optimal cutoff for predicting 6-month mortality for each proposed domain of frailty and to evaluate the sensitivity, specificity, positive predictive value (PPV), negative predictive value (NPV), and accuracy of that cutoff. Each domain of frailty was represented by a single measure with the exception of nutritional status. The two components of nutritional status were evaluated separately for the ROC curve analysis to determine their individual cutoffs and combined for all further analysis. The cutoffs for each domain were used to determine the total impaired domains (the total number of domains that a dog met the cutoff for) and the optimal cutoff from the total impaired domains was used to define overall frailty (whether an individual was frail or non-frail).

**Table 3:** Cox proportional hazard analysis (Population 1).

Domain/measure	Log rank *p*-value	Log-likelihood	HR	Lower 95% CI	Upper 95% CI
Nutritional status	0.00007	5.83	7.04	2.3	21.6
Exhaustion	0.005	3.02	5.46	1.46	20.49
Mobility	0.004	3.61	7.38	1.48	36.71
Muscle condition	0.01	2.41	4.25	1.23	14.74
Social activity levels	0.005	3.41	4.35	1.42	13.32
Total impaired domains	0.00000003	11.2	2.7 per unit	1.78	4.09
Overall frailty	0.001	8.49	8.014	1.75	36.68

Results of the Cox proportional hazard analysis for each domain of frailty (nutritional status, exhaustion, mobility, muscle condition, and social activity levels), total impaired domains, and overall frailty for dogs in population 1. The results include the *p*-value for each measure, the log-likelihood, the hazard ratio (HR) and the 95% confidence interval (CI) of the HR. For each domain of frailty, dogs were assigned to one of two groups based on whether they met the criteria to be considered impaired within that domain or not. The HR represents the difference in risk of 6-month mortality between these two groups. The total impaired domains was determined based on the total number of domains that a dog met the criteria for, which resulted in a score ranging from 0 to 5. The HR for the total impaired domains represents the difference in risk of 6-month mortality with each point increase in total impaired domains. For overall frailty, dogs were assigned to one of two groups (frail or non-frail) based on their total impaired domains (dogs with a total impaired domains of >1 were considered overall frail). Statistical significance was defined as a *p* < 0.05.

### 3.2 Phase 2: Evaluation of the frailty phenotype

Of the 198 dogs in population 2, 49.4% were females (97 spayed and 1 intact), and 50.5% were males (95 neutered and 5 intact). The median age at Time 0 was 12.0 years (range 9.4–16.5 years) with 132 (66.7%) of the dogs’ ages being reported as known, and 66 (33.3%) reported as an estimated age. Of the 198 dogs, 87 (43.9%) were mixed breed and 111 (56%) were reported as purebred dogs. Represented breeds included Beagle (9), Yorkshire Terrier (8), Labrador retriever (7), Golden retriever (6), dachshund (5), Siberian Husky (5), Chihuahua (5), Jack Russell Terrier (4), Border Collie (4), Shih Tzu (4), Bichon Frise (4), Cavalier King Charles Spaniel (3), Miniature Schnauzer (3), Maltese (3), West Highland White Terrier (2), American Staffordshire Terrier (2), Cocker Spaniel (2), Boston Terrier (2), Miniature Australian Shepherd (2), Shetland Sheepdog (2), Toy Poodle (2), Basset Hound (2), Weimaraner (2), German Shepherd (2), Great Dane (2), and one each of the following breeds: Norwich Terrier, Doberman Pinscher, Miniature Poodle, Airedale Terrier, Pekingese, Greyhound, Coton de Tulear, Standard Poodle, Swedish Vallhund, Havanese, Rat Terrier, Plott Hound, Pembroke Welsh corgi, Australian Shepherd, Alaskan Malamute, English Springer Spaniel, Pomeranian, German Shorthaired Pointer, and Samoyed (Table 1).

Of the 198 dogs, 55 (27.7%) died by the 6-month end point of the study. Causes of death were known for 53 of those dogs (96.3%) and included euthanasia (45 dogs; 84.9%) due to progressive neoplasia (15), congestive heart failure (9), mobility issues (6), inappetence (5), respiratory difficulties (4), seizures (2), renal failure (2), pain (1), and for unknown reasons (1); and natural causes (8 dogs; 15.1%) due to progressive neoplasia (2), congestive heart failure (2), renal failure (1), foreign body obstruction (1), protein losing enteropathy (1), and unknown cause (1).

Kaplan–Meier survival curve and Cox proportional hazard analyses were performed using the original measures and cutoffs defined in phase 1 of the study (Table 4). Measures that performed differently than expected were analyzed in more depth to determine their appropriateness for continued use in the study or whether their alternative measure performed better. Receiver operating characteristic curve analyses using 5-fold cross validation were performed for each domain as well as for each alternative measure for all of the domains. The optimal cutoff matched the cutoffs for population 1 in three instances (body condition score, appetite and mobility). For social activity, the optimal cutoff for the initial measure matched the cutoff for population 1; however, the alternative measure (which asked owners how many days of the week their dog was playful) had a better sensitivity and specificity and so replaced the original question in the final analysis. For muscle condition, a cutoff based on the sum of all muscle groups evaluated was found to be a better measure than evaluating the epaxial muscles alone. For body condition score, while the optimal cutoff remained unchanged, there was a clear correlation between mortality and over-conditioning and so the cutoff was adjusted to best account for both over and under-conditioning, rather than just under-conditioning. The cutoff for overall frailty was also adjusted based on ROC curve analysis such that dogs in population 2 were considered overall frail if they were impaired in three or more domains instead of two.

**Table 4:** Pre-adjusted cox proportional hazard analysis (Population 2).

Domain/Measure	Log rank *p*-value	Log-likelihood	HR	Lower 95% CI	Upper 95% CI
Nutritional status	0.013	1.08	1.94	1.14	3.36
Exhaustion	0.001	5.89	2.55	1.44	4.77
Mobility	0.303	1.62	1.32	0.76	2.25
Muscle condition	0.61	0.13	1.19	0.58	2.22
Social activity levels	0.0076	3.19	2.05	1.18	3.5
Total impaired domains	0.0004	5.09	1.35 per unit	1.13	1.62
Overall frailty	0.0019	2.47	2.44	1.36	4.36

Results of the Cox proportional hazard analysis for each domain of frailty (nutritional status, exhaustion, mobility, muscle condition, and social activity levels), total impaired domains, and overall frailty for dogs in population 2 using the original measures and cutoffs proposed from Phase 1 of the study. The results include the *p*-value for each measure, the log-likelihood, the hazard ratio (HR) and the 95% confidence interval (CI) of the HR. For each domain of frailty, dogs were assigned to one of two groups based on whether they met the criteria to be considered impaired within that domain or not. The HR represents the difference in risk of 6-month mortality between these two groups. The total impaired domains was determined based on the total number of frailty domains that a dog met the criteria for, which resulted in a score ranging from 0 to 5. The HR for total impaired domains represents the difference in risk of 6-month mortality with each point increase in the number of impaired domains. For overall frailty, dogs were assigned to one of two groups (frail or non-frail) based on their total impaired domains (dogs with a total impaired domains of >2 were considered overall frail). Statistical significance was defined as a *p* < 0.05.

**Figure 2:**
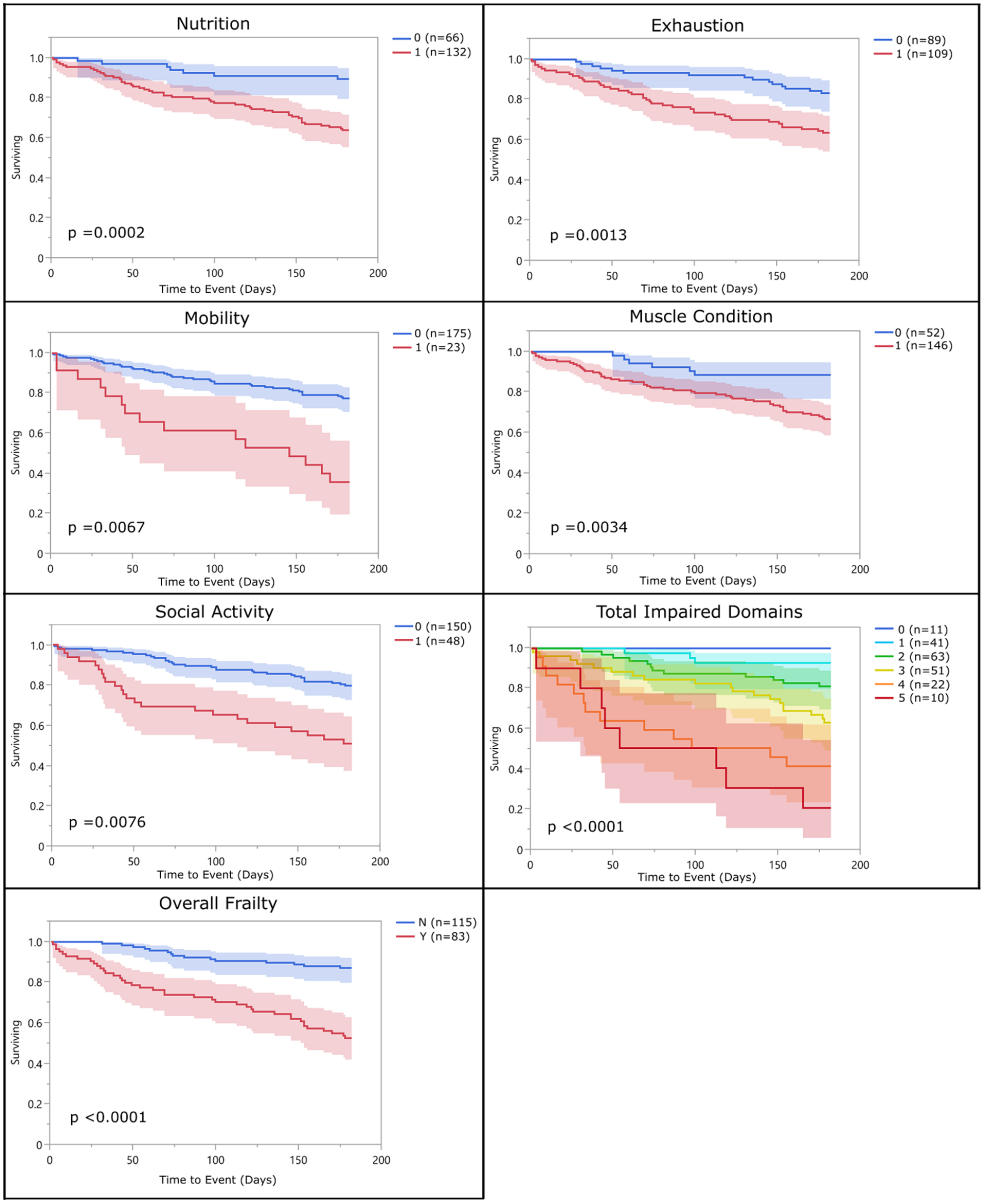
Survival curve by domain (population 2): population 2 survival curves representing the 6-month survival for each frailty domain, total impaired domains, and overall frailty. For each of the frailty domains (nutrition, exhaustion, mobility, muscle condition, and social activity), the blue line (0) represents the dogs who did not meet the criteria to be considered impaired within that domain and the red line (1) represents the dogs who did. Each dog was assigned a total impaired domains score based on the number of domains they met the criteria for (range 0–5). Dogs were classified as overall frail (Y) if they met the criteria for 3 or more domains, and non-frail (N) if they met the criteria for two or fewer of the domains.

With the above adjustments in the frailty scoring, sensitivity for each domain ranged from 16 to 89% and specificity ranged from 32 to 94% (Table 5; Supplementary Data Sheet 3). All domains were significantly associated with 6-month mortality (*p* < 0.05), with hazard ratios ranging from 2.55 to 3.99 (Figure 2; Table 6). Total impaired domains was also significantly associated with 6-month mortality (*p* < 0.0001) with a hazard ratio of 2.12 per unit (95% CI, 1.9–2.65) (Figure 2; Table 6). Overall frailty was also significantly associated with 6-month mortality (*p* < 0.0001) with a hazard ratio of 4.71 (95% CI, 2.66–8.8) (Figure 2 and Table 6) and predicted 6-month mortality with a sensitivity of 73% and specificity of 70% (Table 5). All relationships remained significant even when adjusting for age, weight, breed, and sex. Only breed (purebred vs. mixed breed) remained significant when evaluated alongside all covariates with purebred dogs having a 1.85 times higher mortality rate than mixed breed dogs (95% CI, 1.04–3.31). All covariates were also evaluated with and without frailty and compared to frailty on its own. Models which included frailty had a higher log-likelihood which was supportive of a better fit for predicting mortality (Table 7).

**Table 5:** Receiver operating characteristic curve analysis with 5-fold cross validation (Population 2).

Domain/measure	Optimal cutoff	Average AUC	Average validation AUC	Average sensitivity	Average specificity	PPV	NPV	Accuracy
Nutritional status (body condition category)	2	0.63	0.64	71%	53%	0.37	0.82	0.56
Nutritional status (appetite)	3	0.64	0.63	51%	75%	0.44	0.8	0.68
Exhaustion	2	0.68	0.69	56%	69%	0.42	0.81	0.65
Mobility	7	0.62	0.65	38%	85%	0.6	0.78	0.72
Muscle condition	3	0.62	0.63	82%	41%	0.36	0.86	0.53
Social activity levels	2	0.64	0.66	43%	85%	0.54	0.79	0.73
Total impaired domains	3	0.78	0.79	68%	75%	0.53	0.86	0.73
Overall frailty	1	0.71		73%	70%	0.48	0.87	0.71

Results of the average receiver operating characteristic (ROC) curve analyses using 5-fold cross validation for population 2. The ROC curve analyses were performed to reaffirm the optimal cutoff for predicting 6-month mortality for each proposed domain of frailty and to evaluate the sensitivity, specificity, positive predictive value (PPV), negative predictive value (NPV), and accuracy of that cutoff. Each domain of frailty was represented by a single measure with the exception of nutritional status. The two components of nutritional status were evaluated separately for the ROC curve analysis to determine their individual cutoffs and combined for all further analyses. The cutoffs for each domain were used to determine the total impaired domains (the total number of domains that a dog met the cutoff for) which was also evaluated using a 5-fold cross validation, and the optimal cutoff from the total impaired domains was used to define overall frailty (whether an individual was frail or non-frail) which was evaluated on the entire dataset.

**Table 6:** Cox proportional hazard analysis (Population 2).

Domain/Measure	Log rank *p*-value	Log-Likelihood	HR	Lower 95% CI	Upper 95% CI
Nutritional status	0.0018	8.05	3.99	1.93	9.67
Exhaustion	0.001	5.34	2.55	1.44	4.77
Mobility	<0.0001	8.02	3.9	2.11	7
Muscle condition	0.0034	5.15	3.3	1.53	8.6
Social activity levels	<0.0001	7.996	3.13	1.82	5.33
Total impaired domains	<0.0001	22.1	2.12 per unit	1.7	2.65
Overall frailty	<0.0001	15.1	4.71	2.66	8.8

Results of the Cox proportional hazard analysis for each domain of frailty (nutritional status, exhaustion, mobility, muscle condition, and social activity levels), total impaired domains, and overall frailty for dogs in population 2. The results include the *p*-value for each measure, the hazard ratio (HR) and the 95% confidence interval (CI) of the HR. For each domain of frailty, dogs were assigned to one of two groups based on whether they met the criteria to be considered impaired within that domain or not. The HR represents the difference in risk of 6-month mortality between these two groups. The total impaired domains was determined based on the total number of domains that a dog met the criteria for which resulted in a score ranging from 0 to 5. The HR for total impaired domains represents the difference in risk of 6-month mortality with each point increase in total impaired domains. for overall frailty, dogs were assigned to one of two groups (frail or non-frail) based on their total impaired domains (dogs with a total impaired domain of >2 were considered overall frail). Statistical significance was defined as a *p* < 0.05.

**Table 7:** Cox proportional hazard analysis with covariate adjustments (Population 2).

Covariate adjusted for	Effect	LogWorth	*p*-value	HR	Lower 95% CI	Upper 95% CI
Unadjusted	Frailty	7.428	<0.0001	4.71	2.66	8.8
Age only	Frailty	6.981	<0.0001	4.64	2.59	8.78
	Age	0.086	0.82	1.01 (Older)	0.87	1.19
Breed only	Frailty	7.342	<0.0001	4.67	2.63	8.73
	Breed	1.263	0.055	1.71 (Purebred)	0.98	3.01
Sex only	Frailty	7.41	<0.0001	4.7	2.65	8.79
	Sex	0.134	0.73	1.1 (Male)	0.65	1.86
Weight only	Frailty	7.097	<0.0001	4.6	2.58	8.62
	Weight	0.196	0.637	1	0.98	1.02
Age breed sex and weight	Frailty	5.988	<0.0001	4.22	2.33	8.05
	Age	0.428	0.37	1.08 (Older)	0.91	1.29
	Breed	1.479	0.033	1.85 (Purebed)	1.04	3.31
	Sex	0.081	0.83	1.06 (Male)	0.62	1.81
	Weight	0.584	0.26	1.01	0.99	1.03
Age breed sex and weight without frailty	Age	1.577	0.027	1.21 (Older)	1.02	1.43
	Breed	1.763	0.017	1.97 (Purebred)	1.11	3.51
	Sex	0.165	0.684	1.12 (Male)	0.66	1.90
	Weight	1.634	0.023	1.03	1.00	1.05
Log-likelihood	Frailty only	15.14				
	All covariates including frailty	17.5				
	All covariates without frailty	5.57				

Results of the Cox proportional hazard analysis for overall frailty when accounting for different covariates (age, breed, sex, weight) and all covariates together. Reported results include each of the covariates LogWorth within the model, their *p*-value, the hazard ratio (HR) and the 95% confidence interval (CI). The log-likelihood for overall frailty, all covariates including frailty, and all covariates without frailty are included at the bottom for comparison. Statistical significance was defined as a *p* < 0.05.

**Figure 3:**
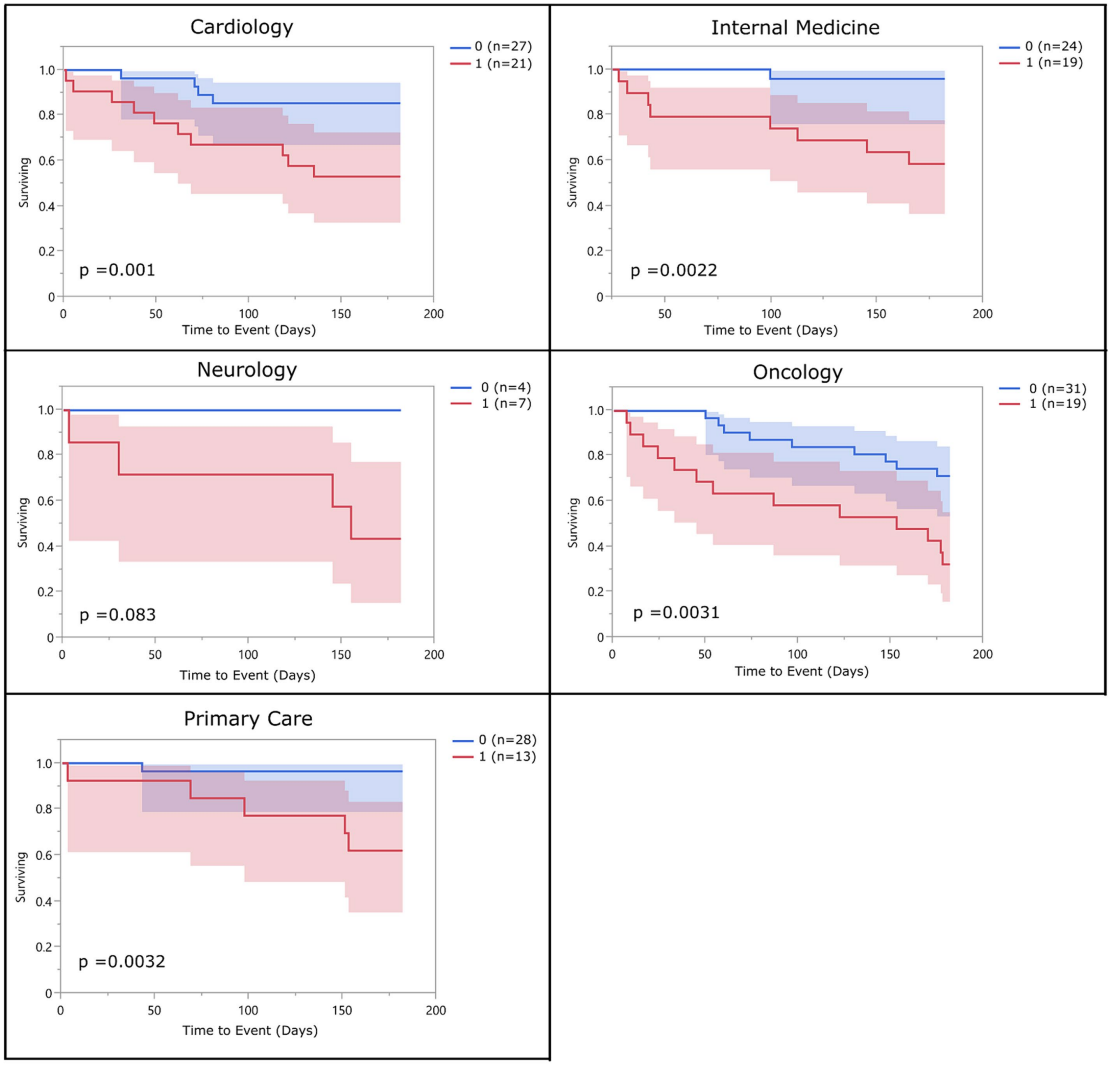
Survival curve by service (population 2): population 2 survival curves representing the 6-month survival for overall frail dogs broken into the different specialty services within the hospital. The blue line (0) represents the non-frail dogs (total impaired domains <3), and the red line (1) represents the overall frail dogs (total impaired domains ≥3).

There were no significant differences in risk of 6-month mortality between different hospital services with the exception of Oncology which had a significantly higher rate of mortality than all other services. The hazard ratio for overall frailty within each service ranged between 2.36 (oncology) and 12.46 (internal medicine) (Table 8). Survival curves were evaluated for each service. In all services, the survival curves of frail and non-frail groups visibly diverged over time. Moreover, survival curves were statistically significant for patients enrolled from the cardiology, oncology, internal medicine, and primary care services. The number of dogs enrolled from the neurology and rehabilitation services were not high enough to reach significance (Figure 3).

**Table 8:** Cox proportional hazard analysis by specialty (Population 2).

Domain/measure	Log rank *p*-value	Log likelihood	HR	Lower 95% CI	Upper 95% CI	Population	Mortality Rate
Cardiology	0.012	3.13	4.02	1.34	14.65	48	14/48 (29.1%)
Internal medicine	0.0018	4.85	12.46	2.28	231.21	43	9/43 (20.9%)
Primary care	0.0055	3.85	12.38	1.99	237.36	41	6/41 (14.6%)
Oncology	0.005	3.93	3.36	1.45	8.16	50	22/50 (44%)

Results of the cox proportional hazard analysis for overall frailty within each hospital service. The results include the p-value for each measure, the log likelihood, the hazard ratio (HR) and the 95% confidence interval (CI) of the HR. Within each service, dogs were defined as either frail or non-frail based upon their total impaired domains. The HR represents the difference in risk of 6-month mortality between the frail and non-frail groups. The population number refers to the total number of patients who were enrolled through that service. The mortality rate is the number of patients from each service that passed away within 6-months of enrollment. Statistical significance was defined as a *p* < 0.05.

## 4 Discussion

Despite the increasing understanding of the importance of frailty on healthy aging in people and the increasing interest in its potential role in the veterinary field, few studies evaluating frailty in our canine companions have been conducted. The goal of this study was to develop a measure of frailty in dogs which was based upon the groundwork laid by previous human and canine frailty studies; we aimed to create and refine a measure that could be deployed as a rapid screen, would have notable clinical relevance to act as a strong tool for clinical decision making, and could act as an early indicator of risk. The results of this study indicate that the final frailty phenotype is statistically associated with short term mortality in older dogs regardless of age, sex, breed, and weight. Dogs who were overall frail were just under five times more likely to die in the 6-month follow-up period than dogs classified as non-frail, and that risk of mortality increased by 2.12 times for each one-point increase in the total impaired domains.

While we attempted to match the domains of frailty defined by previous studies in humans and dogs, this was not always feasible. One measure commonly used to represent nutritional status was unintentional weight loss. While we had the ability to measure weight change in both populations of the study, we found that this was not a reliable measure for unintentional weight loss. Many of the dogs in both populations were on weight loss or weight gain diets (>20% in population 2) which could have confounded these results. In addition, due to the smaller weights of our patients on average compared with people, small variations in weight, which can occur with the use of different scales, may result in falsely elevated weight variations. Weight loss is also often considered a late indicator of frailty which makes it a less useful tool for predicting early risk (19). We therefore focused on appetite and body condition as alternative indicators of nutritional status. In population 1, a low body condition was found to be significantly related to 6-month mortality; however, the effect of a high body condition score could not be fully evaluated due to the low incidence in this population. In population 2 both a low body condition score and a high body condition score were associated with mortality. This is consistent with research on frailty in older women which found that obesity was positively correlated with frailty in that population (20). Similarly, walking speed was measured in the dogs in population 1; however, it was not significantly associated with mortality in this population (unreported data) and was therefore excluded from the analysis.

Walking speed was included in the study conducted by Lemaréchal et al. (14) but was performed on a limited set of dog breeds. We evaluated walking speed on and off leash using senior dogs enrolled from the same study as were enrolled in phase 1 and found that on-leash walking speed was affected by the handler (21) which may have important implications for its accuracy as a measure of true individual walking speed. Of additional concern was whether walking speed could feasibly be assessed in all patients. Patients being exercise restricted or on oxygen support may not be able to safely undergo an evaluation of walking speed which would limit the use of our measure in these patients. Further validation of a walking speed test that is robust across multiple breeds and clinic settings is still needed. For most older dogs, walking speed may be a viable measure as part of a frailty phenotype; however, due to the limitations noted above, it did not meet the goals set for this study. This measure was instead replaced with an owner questionnaire regarding mobility. We considered this a reasonable substitute given the general assumption that dogs with greater mobility impairments are likely to exhibit a slower overall gait and our intent was to develop a rapid screening tool. An owner questionnaire is additionally beneficial in that the results are not impacted by the adrenalin or excitement of being at a hospital setting. However, this alteration of the measure does limit comparison with other canine frailty measures that have utilized walking speed, and deviates further from the human frailty phenotype.

The defined frailty measure performed similarly within all evaluated specialties. However, the hazard ratios varied notably between different services with oncology having the lowest hazard ratio for overall frailty and internal medicine and primary care having the highest. We speculate that this difference is due to the proportion of terminal illness within the populations. In the oncology service the proportion of dogs with terminal disease is very high making frailty a less sensitive measure than for those recruited from the primary care or internal medicine service which likely encompass a broader range of health statuses including healthy geriatric dogs. The sensitivity, specificity and accuracy of prediction of 6-month mortality in population 2 was 73, 70 and 71%, respectively, with a positive predictive value of 48% and negative predictive value of 87%. Ideally a screening tool would have a higher positive predictive value but the high negative predictive value supports a finding of non-frail using this tool.

When evaluating all covariates (age, weight, and breed) with or without frailty included in the model for population 2, it becomes clear that while these covariates may have varying degrees of association with mortality, frailty was the most important predictor of 6-month mortality. This can be seen most clearly when comparing the log-likelihood between the covariates without frailty (5.57) to frailty alone (15.14). Interestingly, in population 2 of the study, no covariates aside from frailty were significantly associated with mortality with the exception of breed. Purebred dogs in population 2 were found to have a 1.85 times higher mortality rate than mixed breed dogs. This could support the concept of hybrid vigor and the assumption that mixed breed dogs are less prone to disease and are generally healthier than purebred dogs. Many studies have looked into the effect of inbreeding on health in a number of both plant and animal species and there is increasing evidence that supports that inbreeding is associated with greater risk of morbidity (22, 23). Further research is still needed in regards to how breed and inbreeding impacts health and in particular, what role it may play on frailty.

In population 1, the optimal cutoff for overall frailty was identified to be dogs meeting the criteria for two or more domains. This was based on the results of the ROC curve analysis, and is consistent with previous studies of frailty in dogs that used the same cutoff (10). In contrast, the optimal cutoff for population 2 was statistically found to be three or more domains. This more closely parallels studies of the frailty phenotype in people and a recent study of frailty in dogs where frailty was defined as meeting the criteria for 3 or more domains and includes the addition of pre-frailty as a classification for people or dogs meeting only 1 or 2 criteria (2, 7, 14, 20). We feel that this adjustment supports the ability to use the updated measure for earlier detection of frailty or risk of frailty which will allow for better identification and intervention for at-risk dogs. While pre-frailty is an important concept particularly as it relates to risk of becoming frail and preventing frailty, we were unable to fully evaluate it in our population. Phase 1 did not have enough complete data to accurately distinguish a pre-frail category nor evaluate frailty in a truly longitudinal fashion and Phase 2 only measured frailty at a single time point. Fried et al. (2) defined the pre-frail state as being those individuals at increased risk of becoming frail. They did this by comparing frailty at the initial timepoint and a follow up time point which showed that individuals with 1 or 2 impaired domains had a greater incidence of being frail at the follow-up time point than individuals with no impaired domains. Future longitudinal studies could be useful to help determine if a pre-frail status is appropriate for use with this study’s defined measures. While the goal of this study was to define a binary state (non-frail or frail) it should be noted that the total impaired domain measure was also predictive of 6-month mortality and that the risk of mortality was cumulative. This suggests that in addition to using this tool to identify a dog as frail, the total impaired domains may also be useful towards sub-classifying the severity of a dog’s frailty, further expanding upon the utility of this tool.

Limitations to this study include the lack of complete data available for all domains for all patients in population 1 and the adjustments to new measures which prevented re-evaluating the prior data using the new cutoffs. The ROC curve analyses were also used to evaluate ordinal data; therefore, sensitivity and specificity findings should be evaluated cautiously with this in mind. This was one reason that the ROC curve analyses were used to support previously identified cutoffs rather than being the primary analysis for the study and emphasizes the importance of evaluating the data using multiple different models. The use of Cox Proportional Hazard models requires that covariates remain constant over time. This was an assumption made for our data set, without specific confirmatory testing. Cox Proportional Hazard models are commonly used to evaluate survival data in medicine though the issue of time as a variable is a frequently recognized concern. In addition, the finite nature of the data adds uncertainty to the robustness of the results when applied in different settings. Certain measures within the proposed phenotype (BCS and MCS) rely on evaluations which are subjective, semi-quantitative measures and may be impacted by interrater variability. Studies evaluating measures of body condition scoring have shown a high level of agreement between different trained individuals (24). Whereas studies on interrater agreement in muscle condition scoring have shown moderate agreement, with the greatest agreement for dogs with normal muscle mass and dogs with severe muscle loss (25).

For phase 2 of the study, it was decided to use a cutoff of 10 years of age instead of using the last 25% of expected lifespan that was used for Phase 1 in order to assess performance of the frailty phenotype screening tool in a broad population of older dogs. Frailty, while being closely linked to chronologic age, should by definition be independent of age. A limitation of this method of selection includes the potential for involuntary exclusion of giant breed dogs. We tried to offset this by adjusting the age cutoff for giant breed dogs to 9 years of age. Despite this, only two giant breed dogs were ultimately enrolled in the study. While we suspect the measure will work similarly for these dogs, the conclusions of this study should be applied cautiously within this population.

Our study also did not include biological or environmental variables within the model. This was partly intentional in that we wanted to determine how well our frailty phenotype worked as a screening tool in dogs irrespective of their different comorbidities. By enrolling dogs from different services of the hospital without limitations, we were able to see that the measure performed similarly across all populations of dogs. However, because we did not require any baseline diagnostic testing by the services, we could not adjust for environmental or biological covariates. Due to the limited number of intact dogs in our population, we were also unable to make any conclusions on differences in performance of the frailty measure in these populations. It is difficult to make a direct comparison of the Hazard Ratio of 4.71 for all cause 6-month mortality with other studies given differences in study design. For example, Lemaréchal et al. (14) reported a Hazard Ratio of 5.86 for 5-year mortality.

The most important aspect of frailty to recognize is that it is a dynamic syndrome, which can worsen but also can improve over time or with intervention. This is key because early recognition of frailty may allow for intervention that could reverse or delay its effects. A review of the natural history and progression of frailty in people suggests that frailty is a vicious cycle which begins when a person develops any component of that cycle. The cycle then propagates and progresses to result in the syndrome of frailty (26). Intervention is most likely to be successful when implemented early in this cycle when only one domain is affected. By the time two more are identified, it may be too late to implement meaningful change (26). This concept is likely present in the syndrome of frailty in dogs and emphasizes the importance of understanding frailty such that interventions can better be implemented to reverse the domains and prevent its consequences. Our understanding of frailty and how we can impact it is an important component of future research. Studies evaluating the effects of rehabilitation, optimal nutritional management, enrichment, and other factors will be important future steps.

As previously discussed, companion dogs are of particular interest in aging research due to the similarities with people in shared environments and lifestyles. They also share many of the same age-related diseases, age associated cognitive impairments, and experience similar physiologic impairments that are important in the pathophysiology and developments of frailty in humans (3, 13). Future studies in dogs that delve further into the complex interplay of biological, systemic, and functional impairments that can lead to frailty will be vital to furthering our understanding of gerontology and geriatrics in dogs and how closely it compares to that of people.

In canine primary care, frailty may be a useful tool to help owners and clinicians decide whether to perform elective anesthetic procedures such as dental prophylaxis and provide one more piece of clinically relevant information when making end of life decisions. It may also be able to act as a useful clinical tool in specialty settings such as helping in the decision-making process when choosing surgical vs. more conservative therapy when treating degenerative intervertebral disc disease, or in selecting a specific therapy for treatment of neoplasia. There is also evidence that measures of frailty may be useful in selecting medications and dose, and in predicting the presence of adverse effects (27). In human studies, measures of frailty have been proposed for a number of clinical decision-making situations. Examples include the use of frailty for guiding decisions on glycemic control in diabetic patients. One study proposed implementing less strict glycemic controls in frail patients who are at greater risk of developing treatment-related adverse effects such as hypoglycemia (28). In cardiology studies, measures of frailty have been proposed as part of patient selection for invasive therapies (29). In oncology studies, frailty was found to be predictive of all-cause and post-operative mortality and was predictive of chemotherapy intolerance and postoperative complications (30). Similar uses for frailty measures in canine patients may be possible and further studies evaluating its effectiveness as a clinical tool are warranted.

In conclusion, development of this frailty screening tool could allow for early risk identification and intervention and alert the clinician to perform a more detailed clinical assessment to identify underlying comorbidities contributing to the development of frailty. While further work needs to be done to determine its utility to predict response to therapy, it can in its current form be used to aid in clinical decision making when evaluating potential tolerance to different therapies, and as an aid for end-of-life decisions. It also opens the door for further research into using frailty screening measures to guide clinical decision making, and in evaluating targeted therapies aimed at reversing frailty in dogs.

## Data Availability

The original contributions presented in the study are included in the article/Supplementary material, further inquiries can be directed to the corresponding author.

## Footnotes

^1^ https://arriveguidelines.org

## Supplementary material

Supplementary Figure S1 Receiver operating characteristic curve analysis for all domains, total impaired domains, and overall frailty for population 1.

Supplementary Figure S2 Receiver operating characteristic curve analysis for all domains, total impaired domains, and overall frailty for population 2.
