# Supplementary material for: Establishing a clinically applicable frailty phenotype screening tool for aging dogs
Source: Front Vet Sci. 2024 Sep 25;11:1335463. doi: 10.3389/fvets.2024.1335463 (PMC11465091; doi:10.3389/fvets.2024.1335463)
Supplement: Supplementary Figure S1 — Receiver operating characteristic curve analysis for all domains, total impaired domains, and overall frailty for population 1. [file Data_Sheet_1.PDF]

Frailty Phenotype Questionnaire and Scoring Guide for Population 1

---

1. Nutrition:

- Body Condition Score (1 - 9)
  - ▢ 1 ▢ 2 ▢ 3 ▢ 4 ▢ 5 ▢ 6 ▢ 7 ▢ 8 ▢ 9
- My dog now:
  - ▢ Has an increased appetite compared to 6 months ago
  - ▢ Eats normally (i.e. eats regular food, and finishes in the same amount of time)
  - ▢ Will still eat their regular food but takes longer to finish
  - ▢ Does not want to eat their regular food but will still eat treats or people food
  - ▢ Acts interested in food but walks away from the bowl without eating
  - ▢ Cannot be enticed to eat no matter what you offer

---

2. Social Activity:

- My dog now:
  - ▢ Plays and interacts with the family as much as 6 months ago
  - ▢ Will greet me but is more reluctant to to play than 6 months ago
  - ▢ Shows interest when I get home, but will not get up
  - ▢ Does not show any interest in his/her favorite toy, and will often not show interest in greeting me or family
  - ▢ Shows no interest in me getting home, and is not interested in any stimuli (treats, strangers coming to the house, etc.)

---

3. Exhaustion:

- How often does your dog rest (stop, sit, or lie down) during exercise?
  - ▢ Never
  - ▢ Hardly ever
  - ▢ Occasionally
  - ▢ Frequently
  - ▢ Very frequently

---

4. Mobility:

- On average, how frequently does your dog display weakness or lameness in his/her limbs when walking?

  Weakness/ Lameness is defined as reduced strength in a limb. This can appear as limping (placing less weight on one leg compared to another), increased effort moving the limbs, scuffing of the paws, short strides, or falling.

  - ▢ Not at all
  - ▢ Less than 10% of the time
  - ▢ 10 - 50% of the time
  - ▢ More than 50% of the time

- On average, how frequently does your dog display stiffness in his/her limbs when walking?

  Stiffness is defined as reduced flexing of the joints. This can present as reduced bending of the limbs, shorter strides, or the dog appearing to be "straight-legged".

  - ▢ Not at all
  - ▢ Less than 10% of the time
  - ▢ 10 - 50% of the time
  - ▢ More than 50% of the time

- Does your dog ever make mistakes while walking?

  Mistakes may include scuffing of the paws, stumbling, swinging the limbs abnormally outward or inward such that they cross, a lack of coordination between the limbs, and/or skipping steps.

  ▢ No ▢ Yes

5. Muscle Condition:

- Epaxial Muscle Condition Score (0 - 3)

  ▢ 0 ▢ 1 ▢ 2 ▢ 3

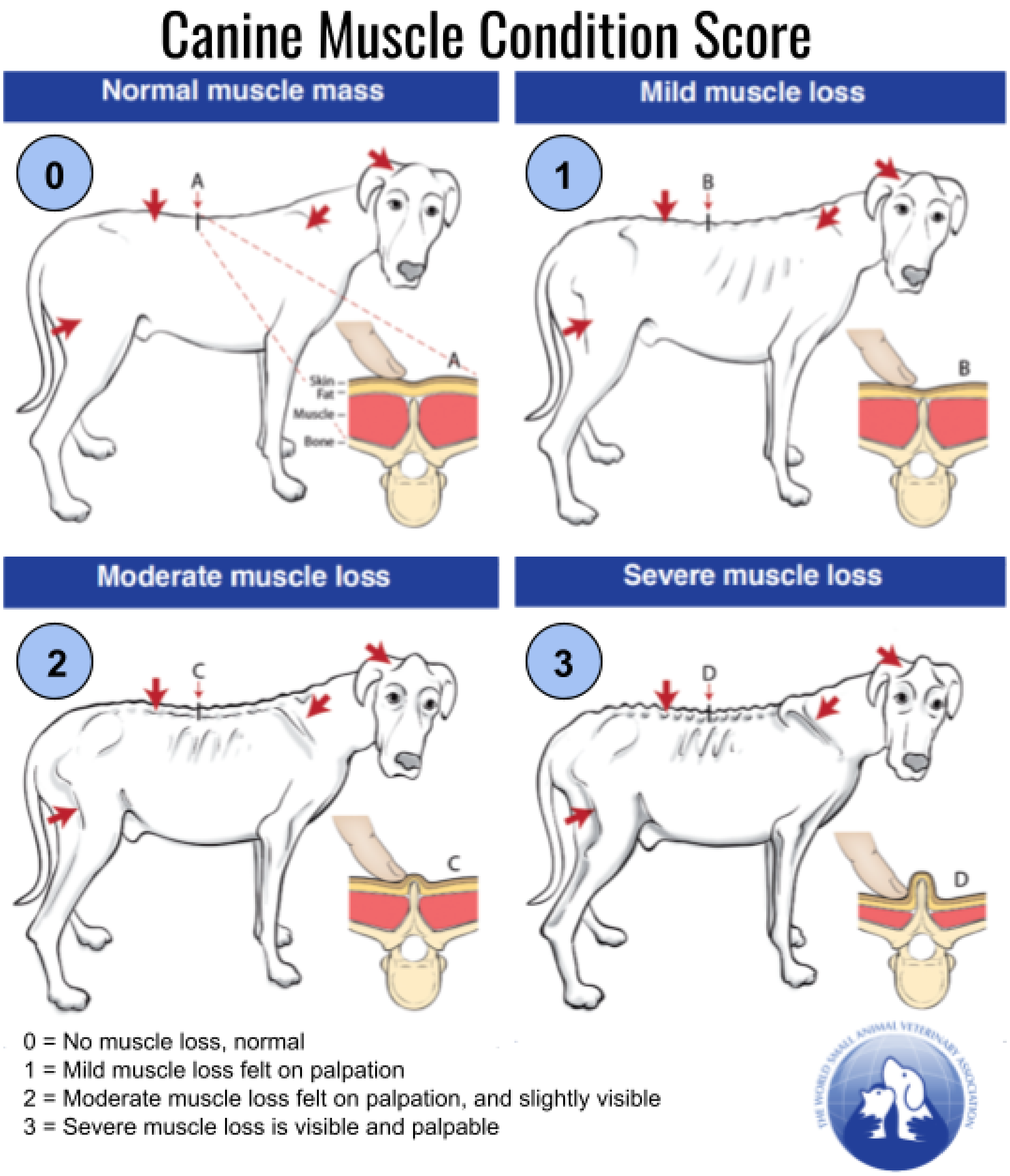

Frailty Phenotype Scoring

Use the following key to complete the summary section and determine the total frailty score

---

1. Nutrition:

- Body Condition Score (1 - 9)

  ▢ 1 ▢ 2 ▢ 3 ▢ 4 ▢ 5 ▢ 6 ▢ 7 ▢ 8 ▢ 9

- My dog now:

  - ▢ Has an increased appetite compared to 6 months ago
  - ▢ Eats normally (i.e. eats regular food, and finishes in the same amount of time)
  - ▢ Will still eat their regular food but takes longer to finish
  - ▢ Does not want to eat their regular food but will still eat treats or people food
  - ▢ Acts interested in food but walks away from the bowl without eating
  - ▢ Cannot be enticed to eat no matter what you offer

---

2. Social Activity:

- My dog now:

  - ▢ Plays and interacts with the family as much as 6 months ago
  - ▢ Will greet me but is more reluctant to to play than 6 months ago
  - ▢ Shows interest when I get home, but will not get up
  - ▢ Does not show any interest in his/her favorite toy, and will often not show interest in greeting me or family
  - ▢ Shows no interest in me getting home, and is not interested in any stimuli (treats, strangers coming to the house, etc.)

---

3. Exhaustion:

- How often does your dog rest (stop, sit, or lie down) during exercise?

  - ▢ Never
  - ▢ Hardly ever
  - ▢ Occasionally
  - ▢ Frequently
  - ▢ Very frequently

---

4. Mobility:

- On average, how frequently does your dog display weakness or lameness in his/her limbs when walking?

  Weakness/ Lameness is defined as reduced strength in a limb. This can appear as limping (placing less weight on one leg compared to another), increased effort moving the limbs, scuffing of the paws, short strides, or falling.

  - ▢ Not at all (0 points)
  - ▢ Less than 10% of the time (1 point)
  - ▢ 10 - 50% of the time (2 points)
  - ▢ More than 50% of the time (3 points)

- On average, how frequently does your dog display stiffness in his/her limbs when walking?

  Stiffness is defined as reduced flexing of the joints. This can present as reduced bending of the limbs, shorter strides, or the dog appearing to be "straight-legged".

  - ▢ Not at all (0 points)
  - ▢ Less than 10% of the time (1 point)
  - ▢ 10 - 50% of the time (2 points)
  - ▢ More than 50% of the time (3 points)

- Does your dog ever make mistakes while walking?

  Mistakes may include scuffing of the paws, stumbling, swinging the limbs abnormally outward or inward such that they cross, a lack of coordination between the limbs, and/or skipping steps.

  ▢ No (0 points) ▢ Yes (1 point)

Sum of Mobility Scores:

▢ 0 ▢ 1 ▢ 2 ▢ 3 ▢ 4 ▢ 5 ▢ 6 ▢ 7

5. Muscle Condition:

- Epaxial Muscle Condition Score (0 - 3)

  ▢ 0 ▢ 1 ▢ 2 ▢ 3

Summary of All Frailty Domains				
For each domain, if any answer fell within the red zones, that domain receives a score of 1<br>If no answers for a domain fell within the red zones, then that domain receives a score of 0<br>Sum the five domains to determine the **Total Frailty Score**				
NUTRITION	SOCIAL ACTIVITY	EXHAUSTION	MOBILITY	MUSCLE CONDITION

**Total Frailty Score**	

Total Frailty Score Interpretation

Non-Frail	Frail
Total Frailty Score 0 or 1	Total Frailty Scores of 2, 3, 4, or 5

Frailty Phenotype Questionnaire and Scoring Guide for Population 2

---

1. Nutrition:

- Body Condition Score (1 - 9)

  ▢ 1 ▢ 2 ▢ 3 ▢ 4 ▢ 5 ▢ 6 ▢ 7 ▢ 8 ▢ 9

- My dog now:

  - ▢ Has an increased appetite compared to 6 months ago
  - ▢ Eats normally (i.e. eats regular food, and finishes in the same amount of time)
  - ▢ Will still eat their regular food but takes longer to finish
  - ▢ Does not want to eat their regular food but will still eat treats or people food
  - ▢ Acts interested in food but walks away from the bowl without eating
  - ▢ Cannot be enticed to eat no matter what you offer

---

2. Social Activity:

- Over the past week, how many days has your dog been playful?

  (No days) ▢ 0 ▢ 1 ▢ 2 ▢ 3 ▢ 4 ▢ 5 ▢ 6 ▢ 7 (Everyday)

---

3. Exhaustion:

- How often does your dog rest (stop, sit, or lie down) during exercise?

  - ▢ Never
  - ▢ Hardly ever
  - ▢ Occasionally
  - ▢ Frequently
  - ▢ Very frequently

---

4. Mobility:

- On average, how frequently does your dog display weakness or lameness in his/her limbs when walking?

  Weakness/ Lameness is defined as reduced strength in a limb. This can appear as limping (placing less weight on one leg compared to another), increased effort moving the limbs, scuffing of the paws, short strides, or falling.

  ▢ Not at all

  ▢ Less than 10% of the time

  ▢ 10 - 50% of the time

  ▢ More than 50% of the time

- On average, how frequently does your dog display stiffness in his/her limbs when walking?

  Stiffness is defined as reduced flexing of the joints. This can present as reduced bending of the limbs, shorter strides, or the dog appearing to be "straight-legged".

  ▢ Not at all

  ▢ Less than 10% of the time

  ▢ 10 - 50% of the time

  ▢ More than 50% of the time

- Does your dog ever make mistakes while walking?

  Mistakes may include scuffing of the paws, stumbling, swinging the limbs abnormally outward or inward such that they cross, a lack of coordination between the limbs, and/or skipping steps.

  ▢ No ▢ Yes

5. Muscle Condition:

- Forelimb Muscle Condition Score (0 - 3)

  ▢ 0 ▢ 1 ▢ 2 ▢ 3

- Hindlimb Muscle Condition Score (0 - 3)

  ▢ 0 ▢ 1 ▢ 2 ▢ 3

- Epaxial Muscle Condition Score (0 - 3)

  ▢ 0 ▢ 1 ▢ 2 ▢ 3

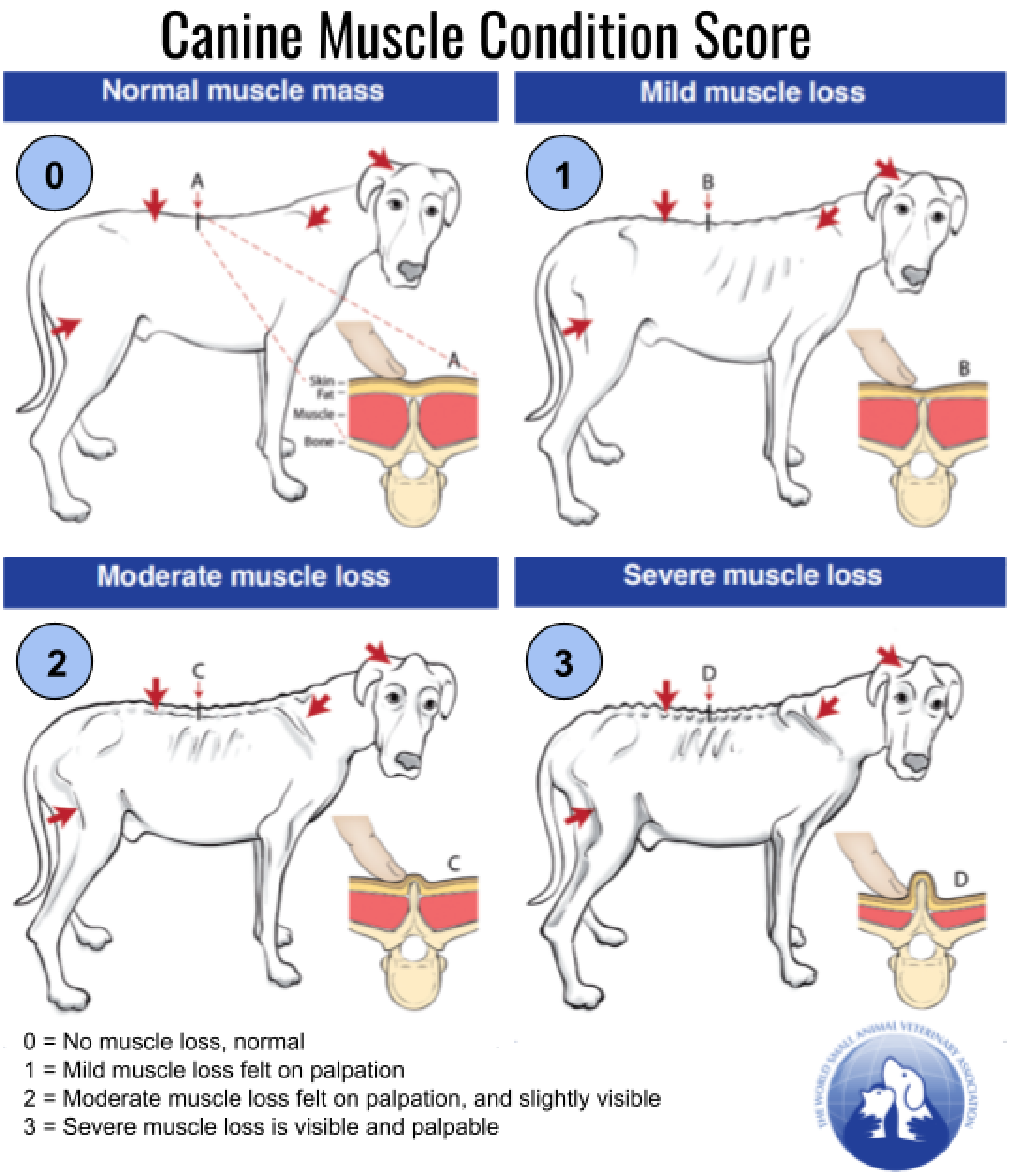

Frailty Phenotype Scoring

Use the following key to complete the summary section and determine the total frailty score

---

1. Nutrition:

- Body Condition Score (1 - 9)

  ▢ 1 ▢ 2 ▢ 3 ▢ 4 ▢ 5 ▢ 6 ▢ 7 ▢ 8 ▢ 9

- My dog now:

  ▢ Has an increased appetite compared to 6 months ago

  ▢ Eats normally (i.e. eats regular food, and finishes in the same amount of time)

  ▢ Will still eat their regular food but takes longer to finish

  ▢ Does not want to eat their regular food but will still eat treats or people food

  ▢ Acts interested in food but walks away from the bowl without eating

  ▢ Cannot be enticed to eat no matter what you offer

---

2. Social Activity:

- Over the past week, how many days has your dog been playful?

  (No days) ▢ 0 ▢ 1 ▢ 2 ▢ 3 ▢ 4 ▢ 5 ▢ 6 ▢ 7 (Everyday)

---

3. Exhaustion:

- How often does your dog rest (stop, sit, or lie down) during exercise?

  ▢ Never

  ▢ Hardly ever

  ▢ Occasionally

  ▢ Frequently

  ▢ Very frequently

---

4. Mobility:

- On average, how frequently does your dog display weakness or lameness in his/her limbs when walking?

  Weakness/ Lameness is defined as reduced strength in a limb. This can appear as limping (placing less weight on one leg compared to another), increased effort moving the limbs, scuffing of the paws, short strides, or falling.

  - ▢ Not at all (0 points)
  - ▢ Less than 10% of the time (1 point)
  - ▢ 10 - 50% of the time (2 points)
  - ▢ More than 50% of the time (3 points)

- On average, how frequently does your dog display stiffness in his/her limbs when walking?

  Stiffness is defined as reduced flexing of the joints. This can present as reduced bending of the limbs, shorter strides, or the dog appearing to be "straight-legged".

  - ▢ Not at all (0 points)
  - ▢ Less than 10% of the time (1 point)
  - ▢ 10 - 50% of the time (2 points)
  - ▢ More than 50% of the time (3 points)

- Does your dog ever make mistakes while walking?

  Mistakes may include scuffing of the paws, stumbling, swinging the limbs abnormally outward or inward such that they cross, a lack of coordination between the limbs, and/or skipping steps.

  ▢ No (0 points) ▢ Yes (1 point)

Sum of Mobility Scores:

▢ 0 ▢ 1 ▢ 2 ▢ 3 ▢ 4 ▢ 5 ▢ 6 ▢ 7

5. Muscle Condition:

- Forelimb Muscle Condition Score (0 - 3)

  ▢ 0 ▢ 1 ▢ 2 ▢ 3

- Hindlimb Muscle Condition Score (0 - 3)

  ▢ 0 ▢ 1 ▢ 2 ▢ 3

- Epaxial Muscle Condition Score (0 - 3)

  ▢ 0 ▢ 1 ▢ 2 ▢ 3

Sum of MCS:

▢ 0 ▢ 1 ▢ 2 ▢ 3 ▢ 4 ▢ 5 ▢ 6 ▢ 7 ▢ 8 ▢ 9

Summary of All Frailty Domains

For each domain, if any answer fell within the red zones, that domain receives a score of 1
If no answers for a domain fell within the red zones, then that domain receives a score of 0

Sum the five domains to determine the **Total Frailty Score**

NUTRITION	SOCIAL ACTIVITY	EXHAUSTION	MOBILITY	MUSCLE CONDITION

**Total Frailty Score**	

Total Frailty Score Interpretation

Non-Frail	Frail
Total Frailty Score of 0, 1, or 2	Total Frailty Scores of 3, 4, or 5
