# Supplementary material for: Establishing a clinically applicable frailty phenotype screening tool for aging dogs
Source: Front Vet Sci. 2024 Sep 25;11:1335463. doi: 10.3389/fvets.2024.1335463 (PMC11465091; doi:10.3389/fvets.2024.1335463)

Receiver Operating Characteristic Curve Analysis (Population 1)

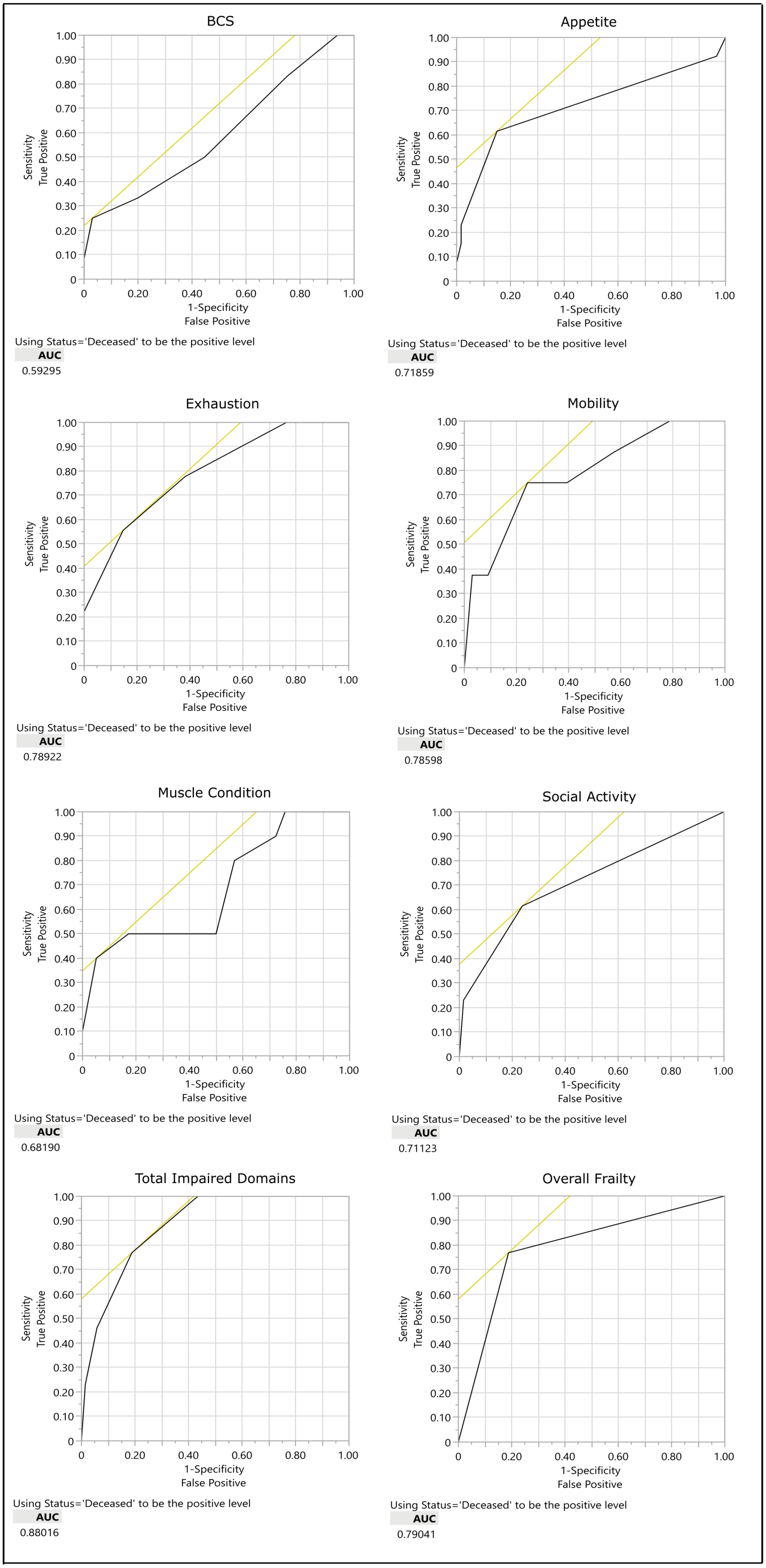

Supplement: Supplementary Figure S2 — Receiver operating characteristic curve analysis for all domains, total impaired domains, and overall frailty for population 2. [file Data_Sheet_2.PDF]
