# Supplementary figures and images for: Establishing a clinically applicable frailty phenotype screening tool for aging dogs
Source: Front Vet Sci. 2024 Sep 25;11:1335463. doi: 10.3389/fvets.2024.1335463 (PMC11465091; doi:10.3389/fvets.2024.1335463)

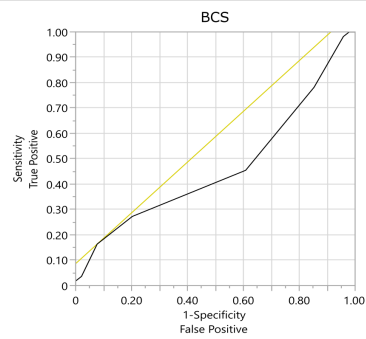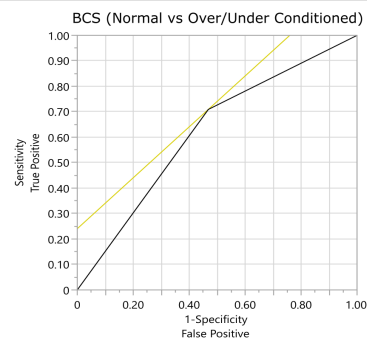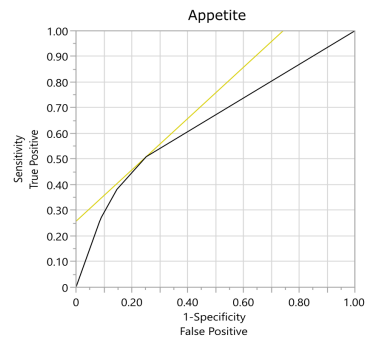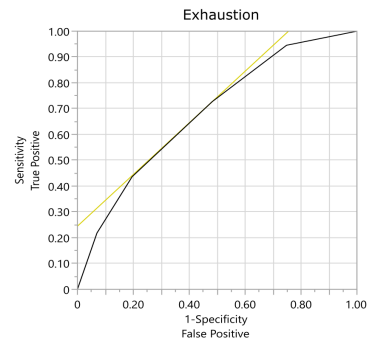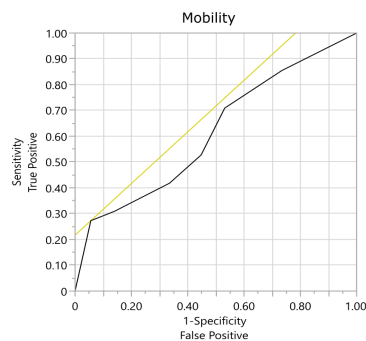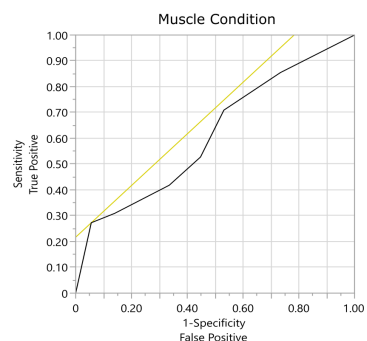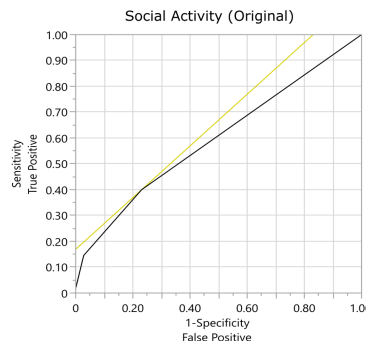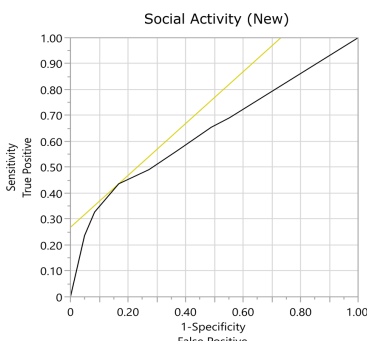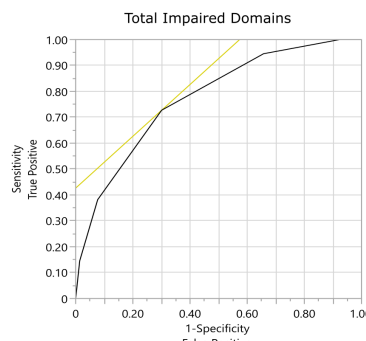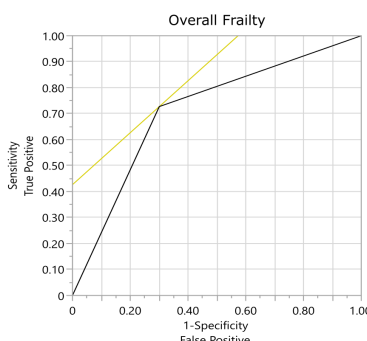

Supplement: Supplementary file 3 [file Data_Sheet_3.PDF]
